# Incisors inclination in relation to lip parameters: a CBCT study

**DOI:** 10.1590/2177-6709.29.6.e2424130.oar

**Published:** 2024-12-16

**Authors:** Majd ELIAS, Sharmin KADKHODAYAN, Camila PACHECO-PEREIRA, Manuel Lagravère VICH

**Affiliations:** 1 University of Alberta, School of Dentistry (Edmonton/Alberta, Canada).

**Keywords:** Cone beam computed tomography, Incisor inclination, Lip protrusion, Lip length, Lip thickness, Tomografia computadorizada de feixe cônico, Inclinação dos incisivos, Protrusão labial, Comprimento dos lábios, Espessura dos lábios

## Abstract

**Introduction:**

With the introduction of Cone Beam Computed Tomography (CBCT) in dentistry, precise measurements are now attainable.

**Objective:**

This study seeks to explore the correlation between incisors inclination and various lip parameters utilizing CBCT technology. Moreover, it aims to assess whether specific incisors inclinations significantly influence lip thickness, length, and position.

**Material and Methods:**

This was a retrospective observational study of available records of orthodontic patients (n=84) aged between 11 and 17.5 years old with pre- and post-treatment CBCT imaging. The 3D Slicer software was used to assess lip parameters and incisors inclinations while adhering to standard CBCT imaging methods. Statistical analysis was conducted to find associations between incisors inclination and lip parameters.

**Results:**

Within certain limits of incisor inclination, lip parameters showed minimal impact. Changes in upper incisor inclination within an average of 5° did not significantly correlate with upper lip parameters. Similarly, changes in lower incisor inclination within an average of 5.6° had no significant effect on lower lip parameters. However, inclination changes of tooth #21 within 5.4° significantly affected upper lip length within 0.35mm.

**Conclusions:**

Lip parameters remained unaffected with specific ranges of incisor inclinations, except for a slight effect on upper lip length with changes in inclination of tooth #21. Clinicians can consider the specific ranges of incisors inclination during treatment planning, especially for patients concerned about lip appearance.

## RESUMO

**Introdução:**

O uso da tomografia computadorizada de feixe cônico (TCFC) na Odontologia possibilitou medições mais precisas.

**Objetivo:**

Este estudo buscou, utilizando a tecnologia TCFC, explorar a correlação entre a inclinação dos incisivos e diferentes parâmetros labiais. Além disso, visou avaliar se inclinações específicas dos incisivos influenciam significativamente na espessura, no comprimento e na posição dos lábios.

**Material e Métodos:**

Esse foi um estudo observacional retrospectivo em registros disponíveis de pacientes ortodônticos (n = 84) com idades entre 11 e 17,5 anos, com imagens de TCFC pré e pós-tratamento. O *software* 3D Slicer foi usado para avaliar os parâmetros labiais e a inclinação dos incisivos, usando métodos padronizados para imagens de TCFC. Uma análise estatística foi realizada para encontrar associações entre a inclinação dos incisivos e os parâmetros labiais.

**Resultados:**

Dentro de certos limites de inclinação dos incisivos, os parâmetros labiais apresentaram impacto mínimo. Mudanças de 5°, em média, na inclinação dos incisivos superiores não se correlacionaram significativamente com os parâmetros do lábio superior. Da mesma forma, mudanças de 5,6°, em média, na inclinação dos incisivos inferiores não tiveram efeito significativo nos parâmetros do lábio inferior. No entanto, mudanças de 5,4° na inclinação no dente #21 afetaram significativamente, em 0,35mm, o comprimento do lábio superior.

**Conclusões:**

Os parâmetros labiais permaneceram inalterados dentro de faixas específicas de inclinações dos incisivos, exceto por um leve efeito no comprimento do lábio superior com mudanças na inclinação do dente #21. Os dentistas devem considerar as faixas específicas de inclinação dos incisivos durante o planejamento do tratamento, especialmente para pacientes preocupados com a aparência dos lábios.

## INTRODUCTION

Lip fullness is considered one of the esthetic components of an attractive face.^1^ Many races view the lack of lip projection as a displeasing sign and as an indication of aging.^2^ ^,^ ^3^ Therefore, the natural growth of the craniofacial complex has been studied to understand its role in changing the shape of the facial profile and soft tissue, including the lips.^4^ The growth of soft tissue occurs in specific growth centers, and predicting the amount and timing of this growth is essential for orthodontists to manipulate soft tissue shape by modifying the hard tissue.^5^

Many patients seek help to reach a more desirable lip appearance.^6^ Since the upper and lower incisors are closely related to the lips, orthodontists are approached for assistance.^7^ Previous studies^8^ ^-^ ^12^ attempted to relate soft tissue changes to changes in incisors inclinations; however, those studies used two-dimensional (2D) radiographic modalities (lateral cephalometric) to analyze the facial soft tissue changes, limiting the results of changes happening in the midsagittal plane.^9^ ^-^ ^12^ When assessing three-dimensional (3D) structures using conventional cephalometric radiographs, inaccuracies may arise due to the inherent limitations of representing such structures on a 2D plane, due to the superimposition of soft and hard tissue structures, particularly when precise quantitative evaluations are necessary.^13^ Based on this context, many studies concluded that predicting the ratio of movement between soft and hard tissue cannot be precisely made.^8^ ^,^ ^14^ ^-^ ^17^

With the introduction of advanced imaging technology to dentistry,^18^ cone-beam computed tomography (CBCT) use increases the possibility of reaching an accurate measurement of the correlation between the movement of the dentoalveolar complex and the facial soft tissue.^19^ ^,^ ^20^ It offers clear advantages in imaging quality, with minimal distortion and the ability to capture crucial anatomical details without overlapping. This technology minimizes the limitations of previous measurement methods that led to the unpredictability of the ratio of movement between hard and soft tissue, by delivering high-resolution 3D images at a cost-effective rate while keeping radiation exposure to a minimum.^13^

There is a limited amount of research on the effect of incisor inclination on lip parameters using advanced imaging technology. Changes in incisor inclination with orthodontic treatment can affect lip posture and competence. Proclined incisors may lead to lip strain, causing the upper lip to appear more stretched and potentially negatively impacting lip esthetics. Therefore, this study aims to determine if there is an effect of inclining the incisors on protruding or retruding the lips. This can inform clinicians about the impact of incisor inclination changes on lip esthetics and will allow for a precise measurement (if any), and tailoring treatment plans to each patient’s unique esthetic preferences and facial features, ultimately optimizing treatment outcomes.

## METHODS

The Research Ethics Board at the University of Alberta in Canada approved this retrospective observational study protocol under number Pro00013379_REN14, addressing the following research question: *“Is there any relation between the inclination of incisors and lip protrusion?”*

**Figure 1:**
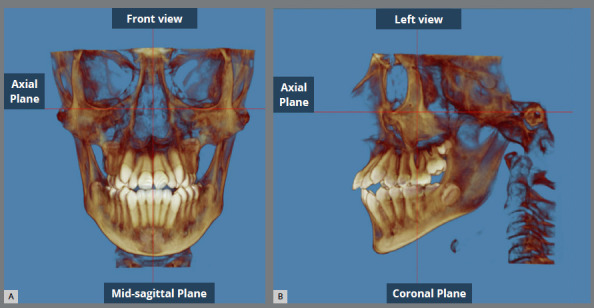
A) Frontal view orientation. B) Lateral view orientation.

The study sample consisted of a pool of available CBCT images from 84 subjects (48 female, and 36 male) between the ages of 11 years and 17.5 years. The subjects had Class I malocclusion with no severe vertical pattern issues before the start of the treatment with self-ligating brackets. This study sample is part of a previous research study that required the use of CBCT scan taken before and after treatment for monitoring. Scans were from the university patients’ records who underwent fixed orthodontic treatment on the permanent teeth between 2011 and 2019. No CBCT images were taken specifically for this study; the analysis of existing images shows the advantages of measurements performed on CBCT axial slices instead of teleradiographs, according to the ALARA principle.^20^ The inclusion criteria were: (1) patients with Class I malocclusion before the start of the treatment; (2) orthodontic treatment with CBCT available from before and after treatment with full fixed self-ligating brackets; (3) natural central and lateral incisors with no prosthodontic treatment. All subjects’ CBCTs were done by the same technician, using a standardized CBCT imaging protocol. The images were taken with patients’ lips in rest position using an i-CAT Classic (Imaging Sciences International, Hatfield, Pa) with a customized field of view of a maximum of 12 in, 300-mm voxel, with the patient in an upright position and in maximum intercuspation. Images were stored in DICOM format. The CBCT scans were taken before treatment (T0) and at the end of the treatment (T1), and were anonymized. A standardized protocol was applied to reorient the images according to head position orientation. To minimize errors, Dolphin Imaging (version 11.95, Dolphin Imaging & Management Solutions, Canoga Park, CA, USA) tool was used by the same researcher, who oriented all images as follows (Table 1 and Fig. 1)^13^:

**Table 1:** Landmark definitions.

Landmarks adopted as reference for the measurements of incisors inclination	
ANS	Anterior nasal spine: the most anterior point of the anterior palatal bone and the floor of the nose cavity, in the midsagittal median point
PNS	Posterior nasal spine: the most posterior point of the posterior palatal bone and the floor of the nose cavity, in the midsagittal median point
Mandibular plane	The plane of the lower border of the mandible. A line passing through Gonion to Menton
CEJ	The junction between enamel and cementum of the tooth
Landmarks adopted as reference for the measurements of lip position	
SN	The point where the nasal septum meets the upper lip in the midsagittal median point
Pog	The most protruded point of the mandible in the midline
E plane	A line passing from the tip of the nose to the tip of the chin
U1-SN	The angle between the long axis of the upper central incisor and the Sella-Nasion
IMPA	The angle formed between the long axis of the lower central incisor and the mandibular plane

1. Frontal view: the axial plane crossed the right and left Orbitale points, and the sagittal plane crossed the anterior nasal spine (ANS).^13^
2. Lateral view: the axial plane was parallel to the Frankfort horizontal plane from Porion in the external auditory meatus to Orbitale in the infraorbital rim on both the right and left side.^13^
3. Occlusal view: the axial plane crossed the posterior nasal spine point located on the frontal view in a vertical axis crossing the naso-frontozygomatic plane.^13^

Measurements were done using 3D Slicer software v. 5.2.2 by two dentists: the first measured each volume twice with randomized repeated measurements two weeks apart, to minimize method error; the second, without access to the previous measurements, repeated the measurement once, to determine the reliability and measurement error of the first two measurements. A standardized Excel (Microsoft Corp, Redmond, USA) document was used to collect data.

**Figure 2:**
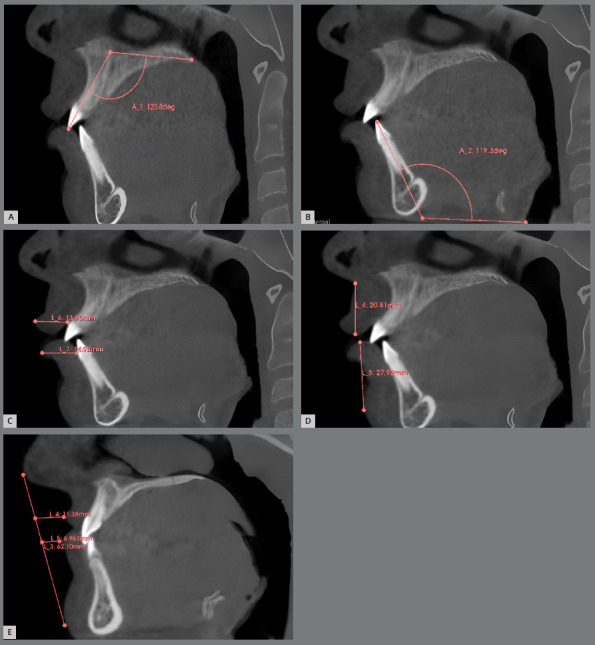
A) Upper incisors inclination. B) Lower incisors inclination. C) Upper and lower lips thickness. D) Upper and lower lips length. E) Upper and lower lips distance to E plane.

The representation of the axial plane was set to cross the most apical point of the cementoenamel Junction (CEJ) of the upper incisors, when measuring the upper lip; and the most apical point of the CEJ of the lower incisors, when measuring the lower lip. This was to provide a better view to measure the lip parameters. The representation of the coronal plane was set to cross the most protruded point of the crown of the lower incisor. This was to provide a better view to locate the midline of each anterior tooth to be measured. The representation of the sagittal plane was set to cross the middle of the tooth to be measured, to provide a better view of the measured tooth (Fig 2).

Incisor inclination: Using the CBCT sagittal plane, the angulation of maxillary incisors was measured in degrees, using the angle formed between the long axis of the tooth (from the incisal edge to the apex at midline) and the maxillary plane from ANS to Posterior Nasal Spine (PNS). The angulation of lower incisors was measured by the angle formed between the long axis of the tooth and the mandibular plane, that was established on the sagittal plane using the line passing through the left Menton and Gonion.

Lip position: The resting lip position was measured at two points on the CBCT sagittal plane.

1. The most protruded and most retruded points on the sagittal plane.
2a. Upper lip: The distance between the Subnasale (SN) and the upper vermillion border was measured in millimeters.
2b. Lower lip: The distance between the soft tissue Pogonion (Pog) and the lower vermillion border was measured in millimeters.
3. Upper and lower lips distance to the E plane. A change of 2mm with respect to the anteroposterior reference plane in the lip was considered clinically significant, following the guidelines determined in previous studies^6^ ^-^ ^11^ done using lateral cephalograms.

For statistical analysis, the means and standard deviations of all the measurements were calculated for the two groups, defined as control and case groups. Intra and inter-reliability of examiners were explored. Statistical analysis was performed using IBM SPSS software to assess the effects of the variables “upper incisor inclination, lower incisor inclination” on “upper lip thickness, upper lip length, upper lip position to E- line, lower lip thickness, lower lip length, lower lip position to E-line”, respectively. The Chi-square test and Student’s *t*-test were used to assess the homogenous distribution of sex and age variables between the control and case groups. A *p*-value was considered significant if less than 0.05. 

## RESULTS

Intra-examiner and inter-examiner reliability for the absolute agreement was assessed using the Cronbach alpha test, and was equal to 0.89 and 0.85, respectively. The baseline measurements for upper and lower incisors inclination at T0 were UI-SN = 97.95° and IMPA = 85.35°.

Table 2 outlines the statistical changes in lip parameters in relation to changes to teeth #11, #21, #31 and #41. Assessments for tooth #11 showed no statistical significance of its inclination within 4.49° on lip thickness (*p*= 0.25), lip length (*p*= 0.1), or lip position (*p*= 0.48). With respect to tooth #21 inclination up to 5.4°, change in lip thickness (*p*= 0.51) and change in lip position (*p*= 0.85) were statistically insignificant, while change in lip length (*p*= 0.02) was statistically significant.

**Table 2:** Statistical details of parameters with respect to teeth #11 (upper right central incisor), #21 (upper left central incisor), #31 (lower left central incisor) and #41 (lower right central incisor).

Correlations						
		t1-t0 of #11	t1-t0 of #21	Upper lip thickness t1-t0	Upper lip length t1-t0	Upper lip position t1-t0
t1-t0 of #11	Pearson correlation	1	0.770	-0.127	-0.179	0.077
t1-t0 of #11	Sig. (2-tailed)		0.000	0.251	0.103	0.484
t1-t0 of #11	n	84	84	84	84	84
t1-t0 of #21	Pearson correlation	0.770	1	-0.073	-0.239	0.021
t1-t0 of #21	Sig. (2-tailed)	0.000		0.512	0.029	0.852
t1-t0 of #21	n	84	84	84	84	84
		t1-t0 of #31	t1-t0 of #41	Lower lip thickness t1-t0	Lower lip length t1-t0	Lower lip position t1-t0
t1-t0 of #31	Pearson correlation	1	0.847	0.150	0.081	-0.024
t1-t0 of #31	Sig. (2-tailed)		0.000	0.173	0.462	0.826
t1-t0 of #31	n	84	84	84	84	84
t1-t0 of #41	Pearson correlation	0.847	1	0.135	0.079	-0.048
t1-t0 of #41	Sig. (2-tailed)	0.000		0.220	0.472	0.662
t1-t0 of #41	n	84	84	84	84	84

Assessments concerning the tooth #31 showed no statistical significance of incisor inclination within 4.33° on lip thickness (*p*= 0.17), lip length (*p*= 0.46) or lip position (P=0.82). With respect to the tooth #41 inclination up to 5.84°, change in lip thickness (*p*= 0.22), lip length (*p*= 0.47) and in lip position (*p*= 0.66) were statistically insignificant.

Table 3 outlines the descriptive statistics for T0 and T1 of the changes in lip parameters in relation to changes to teeth #11, #21, #31, and #41. Notably, increases in upper incisor inclination did not correlate with changes in upper lip thickness, which averaged 13.26mm at T0 and 13.52mm at T1, within the given ranges of 4.49° for #11 and 5.4° for #21. The average thickness of the lower lip was 12.97 mm at T0 and 13.19 mm at T1, indicating that variations in lower incisor inclination, which fell between 4.33° for #31 and 5.84° for #41, did not affect the thickness of the lower lip. Except for the significant influence of #21 inclination (within 5.4°) on upper lip length, which averaged 21.15 mm at T0 and 21.5 mm at T1, lip length remained largely unchanged. Furthermore, no statistically significant associations were discovered between the inclination of the incisor and the location of the upper or lower lips, which averaged -4.34 mm at T0 and -4.93 mm at T1, and -2.79 mm at T0 and -2.71 mm at T1, respectively.

**Table 3:** Descriptive statistic with respect to T0 and T1.

	Minimum	Maximum	Mean	SD
t0 of #11	95.20	130.20	111.6868	6.67142
t1of #11	101.80	154.10	116.1771	8.61643
t1-t0 of #11	-17.50	51.50	4.4905	11.00515
t0 of #21	100.70	124.30	110.8195	5.59063
t1 of #21	102.90	151.40	116.2290	8.34402
t1-t0 of #21	-14.20	40.20	5.4095	8.85436
t0 of #31	85.10	138.90	113.9571	11.04870
t1 of #31	91.20	174.80	118.2927	15.64646
t1-t0 of #31	-30.30	42.50	4.3356	11.66716
t0 of #41	85.60	134.00	112.9878	10.49953
t1 of #41	93.50	167.60	118.8317	14.89776
t1-t0 of #41	-15.50	41.50	5.8439	11.28743
Upper lip thickness t0	9.82	16.71	13.2659	1.87829
Upper lip thickness t1	8.20	18.34	13.5249	1.88928
Upper lip thickness t1-t0	-2.37	3.22	0.2590	1.61865
Upper lip length t0	15.43	27.84	21.1588	2.93299
Upper lip length t1	16.62	26.91	21.5095	2.56748
Upper lip length t1-t0	-3.24	4.17	0.3507	1.62278
Lower lip thickness t0	9.63	17.72	12.9729	2.00851
Lower lip thickness t1	9.28	20.78	13.1978	1.96447
Lower lip thickness t1-t0	-3.34	5.69	0.2249	1.95858
Lower lip length t0	24.01	38.14	30.5966	3.21147
Lower lip length t1	24.05	40.36	32.1712	3.63061
Lower lip length t1-t0	-3.82	6.07	1.5746	2.21836
Upper lip position t0	-12.57	3.93	-4.3483	3.53411
Upper lip position t1	-11.45	2.75	-4.9383	3.26240
Upper lip position t1-t0	-5.83	2.54	-0.5900	1.78709
Lower lip position t0	-10.01	6.48	-2.7968	3.42620
Lower lip position t1	-9.49	5.87	-2.7124	3.79403
Lower lip position t1-t0	-6.04	4.68	0.0844	2.04141

## DISCUSSION

The general population has shown more interest in the importance of facial esthetics, which is a motivating factor for people to seek orthodontic treatment.^21^ While this study established specific degrees of incisor inclination change within which lip parameters remain relatively unaffected, it is essential to recognize the dynamic nature of facial esthetics and individual variations in patient preferences while building a treatment plan for the patient.^22^

This study contributes to the understanding of esthetics by examining how incisors inclination may impact lip position, thickness, and length. Utilizing 3D imaging technology, this study overcomes the limitations of previous research conducted with 2D lateral cephalometric radiographic techniques. By employing CBCT imaging, high-resolution 3D images were obtained, enabling precise measurements, eliminating superimposition, and addressing inconsistencies found in earlier studies that relied on conventional cephalometric radiographs. Moreover, using CBCT technology allowed to view the third plane (transverse) clearly. The present study used all three planes to ensure correct alignment of the 3D scans. This in turn stabilized the landmarks and aided in accurate measurements.

The study conducted in 2022 by Le et al.^6^ found an increase in upper lip thickness following orthodontic treatment. This finding was supported by other studies.^23^ ^,^ ^24^ On the contrary, Hayashida et al.^11^ conducted a study that found a 0.52-mm decrease in lip thickness following orthodontic therapy. There was no mention of the degree of change in tooth inclination in any of these papers. The present study results found no correlation between the change in the inclination of upper incisors and the thickness of the upper lip. Hayashida et al.^11^ found a 2.02-mm increase in thickness of the lower lip, while Lai et al.^23^ found a decrease in lower lip thickness following orthodontic treatment. The present study, similar to Talass et al.^24^, found that a change in the inclination of lower incisors has no effect on the thickness of the lower lip within the limit of 4.33° for tooth #31 and 5.84° for tooth#41 when the lower lip thickness was at an average of 12.97mm at T0 and 13.19mm at T1.

As the lip length influences incisor visibility and has an effect on esthetic perseverance^25^, the present study assesses whether retraction or proclination of incisors has any effect on the length of the lip, which could correlate to the smile line of the patient. A previous study by Le et al.^6^ also showed a relationship between incisor inclinations and changes in the length of the upper lip. It stated that the upper lip length decreased by an average of 1.94mm upon a change in incisor inclinations within 8.35°. However, the present study found no correlation between the change of tooth #11 inclination and the upper lip length within 4.49° of change, and also found that the change in inclination of tooth #21 within the range of 5.4° has an effect on upper lip length with an average length of 21.15mm at T0 and 21.5mm at T1. This is in alignment with the study conducted by Le et al.^6^, in which no correlation was found between the change of lower central incisor inclinations and the length of the lower lip.

This study found no significant correlation between incisor inclination and upper or lower lip position when the average upper lip position at T0 was -4.34mm and at -4.93mm at T1, and the lower lip position average of -2.79mm at T0 and -2.71mm at T1, contradictory to what Le et al.^6^ found during their assessment on Vietnamese population’s lip position after retracting the incisors. This could be due to racial differences, as the present study did not account for any specific race. Le et al.^6^ showed a significant correlation between incisor retraction and lip protrusion, leading to a decrease in an average of 6.75mm in lip prominence after treatment. Bourzgui et al.^7^ found that there was a decrease in lower lip vermilion position in the sagittal plane following orthodontic treatment.

The results suggest that within certain limits of inclination change, the impact on the upper lip is minimal, and the effect on upper and lower lips thickness and position, and lower lip length is absent. These findings have important clinical implications for orthodontic treatment planning and customization based on individual esthetic preferences and facial features.^24^ Therefore, orthodontic treatment plans can potentially be tailored to achieve desired esthetic outcomes without causing adverse effects on lip esthetics, ultimately optimizing treatment results.^25^ ^,^ ^26^

The present study investigated a sample with a specified degree range for upper and lower incisors inclination change, and focused on a specific age group. It mainly concentrated on the effect of incisor inclination on lip parameters, while other factors such as the underlying bony structure of the face, muscular tone, facial fat and collagen composition, genetics and environmental factors, the transversal and anterior-posterior changes related to the treatment and others factors may play a role in changing the lip parameters. However, the sample coming from a university orthodontic clinic in Canada, a country known for multiculturalism and racial diversity, enhances the representativeness of the sample and the broader applicability of the results.

The present findings contribute with valuable insights into the relationship between incisors inclination and lip esthetics. When planning treatment, clinicians should consider specific ranges of incisor inclination changes, especially for patients who are concerned about their lip esthetics. An incisor inclination change of 5 degrees or less appears to have minimal impact on lip parameters such as thickness, length, and position.

Clinicians can integrate this study’s findings with other factors influencing lip position, thereby aiding in the treatment planning for their patients. This includes determining the optimal inclination of the upper and lower central incisors to achieve desired aesthetic facial outcomes concerning the lip parameters. The study does not aim to encourage clinicians to use CBCT for every patient requiring orthodontic treatment to monitor the incisors inclination or lip parameters, and urge them to adhere to the ALARA principle when treating their patients. Lip response to changes in incisors inclination may differ if the baseline measurements changed. It is essential for clinicians to recognize the dynamic nature of facial esthetics and the unique individual variations in patient preferences when building a treatment plan. While the study provides valuable insights into the relationship between incisors inclination and lip esthetics, it’s crucial to consider other factors that may influence lip parameters. These numerical findings contribute with valuable insights into our understanding of the relationship between incisors inclination and lip esthetics, understanding the importance of a comprehensive approach to treatment planning.

The limitations of this study should be considered. The use of existing data and the retrospective nature of the study poses some limitations, such as potential selection bias and the inability to control the confounding variables. Moreover, it was used a sample that is limited to a narrow age range of patients (11 to 17.5 years old), which introduced a limitation in assessing the effects of incisors inclination on lip parameters across different age groups. Finally, the range of incisors inclination of the sample used in this study may not represent the full spectrum of possible changes of inclination after orthodontic treatment.

Future studies could involve a more diverse and extensive sample, considering variations in ethnicities, skeletal patterns, and treatment modalities, such as fixed brackets vs. clear aligner therapy, to identify optimal treatment modalities to achieve the desired lip esthetic. Additionally, longitudinal studies tracking patients over an extended period would provide insights into the stability of the observed changes in lip parameters and their impact on long-term facial esthetics. Furthermore, Investigating the effects of other factors on lip parameters beyond the incisors inclination could be a part of a multifactorial analysis.

## CONCLUSION

At the mentioned baseline angles for upper and lower incisors, lip parameters were not affected by incisor inclination modification within a few specific ranges. Changes in upper incisor inclination showed no correlation to the changes in upper lip thickness or position. Likewise, variations within the specified ranges of teeth #31 and #41 did not have any significant effect on lower lip length, thickness, or position. However, a statistically significant influence of changes in tooth #21 inclination on upper lip length was observed, averaging 0.35mm, which may not hold clinical significance in many cases.
